# Transversus Abdominis Plane Block: An Updated Review of Anatomy and Techniques

**DOI:** 10.1155/2017/8284363

**Published:** 2017-10-31

**Authors:** Hsiao-Chien Tsai, Takayuki Yoshida, Tai-Yuan Chuang, Sheng-Feng Yang, Chuen-Chau Chang, Han-Yun Yao, Yu-Ting Tai, Jui-An Lin, Kung-Yen Chen

**Affiliations:** ^1^Department of Anesthesiology, Taipei Medical University Hospital, Taipei, Taiwan; ^2^Department of Anesthesiology, Kansai Medical University Hospital, Osaka, Japan; ^3^Department of Orthopedics, Wan Fang Hospital, Taipei Medical University, Taipei, Taiwan; ^4^Department of Orthopedics, School of Medicine, College of Medicine, Taipei Medical University, Taipei, Taiwan; ^5^Department of Anesthesiology, Wan Fang Hospital, Taipei Medical University, Taipei, Taiwan; ^6^Health Policy Research Center, Taipei Medical University Hospital, Taipei, Taiwan; ^7^Department of Anesthesiology, School of Medicine, College of Medicine, Taipei Medical University, Taipei, Taiwan

## Abstract

**Purpose of Review:**

Transversus abdominis plane (TAP) block is a regional technique for analgesia of the anterolateral abdominal wall. This review highlights the nomenclature system and recent advances in TAP block techniques and proposes directions for future research.

**Recent Findings:**

Ultrasound guidance is now considered the gold standard in TAP blocks. It is easy to acquire ultrasound images; it can be used in many surgeries involving the anterolateral abdominal wall. However, the efficacy of ultrasound-guided TAP blocks is not consistent, which might be due to the use of different approaches. The choice of technique influences the involved area and block duration. To investigate the actual analgesic effects of TAP blocks, we unified the nomenclature system and clarified the definition of each technique. Although a single-shot TAP block is limited in duration, it is still the candidate of the analgesic standard for abdominal wall surgery because the use of the catheter technique and liposomal bupivacaine may overcome this limitation.

**Summary:**

Ultrasound-guided TAP blocks are commonly used. With the unified nomenclature and the development of catheter technique and/or liposomal local anesthetics, TAP blocks can be applied more appropriately to achieve better pain control.

## 1. Introduction

The transversus abdominis plane (TAP) block was first introduced by Rafi [1] in 2001 as a landmark-guided technique via the triangle of Petit to achieve a field block. It involves the injection of a local anesthetic solution into a plane between the internal oblique muscle and transversus abdominis muscle. Since the thoracolumbar nerves originating from the T6 to L1 spinal roots run into this plane and supply sensory nerves to the anterolateral abdominal wall [2], the local anesthetic spread in this plane can block the neural afferents and provide analgesia to the anterolateral abdominal wall.

With the advancement of ultrasound technology, TAP blocks become technically easier and safer to perform. Thus, there was a surge of interest in TAP blocks as therapeutic adjuncts for analgesia after abdominal surgeries. In the past decade, there has been growing evidence supporting the effectiveness of TAP blocks for a variety of abdominal surgeries, such as cesarean section, hysterectomy, cholecystectomy, colectomy, prostatectomy, and hernia repair [1, 3–9]. Although its analgesic effect covers only somatic pain with short duration [10], single-shot TAP block plays a valuable role in multimodal analgesia. With continuous infusion [11–17] or prolonged-release liposomal local anesthetics [18–22], TAP blocks could overcome the problem of short duration.

In this review, we will describe the relevant anatomy, formulate a nomenclature system to include various approaches, discuss recent advancements in techniques, and detail the possible complications.

## 2. Applied Anatomy

**Figure 1:**
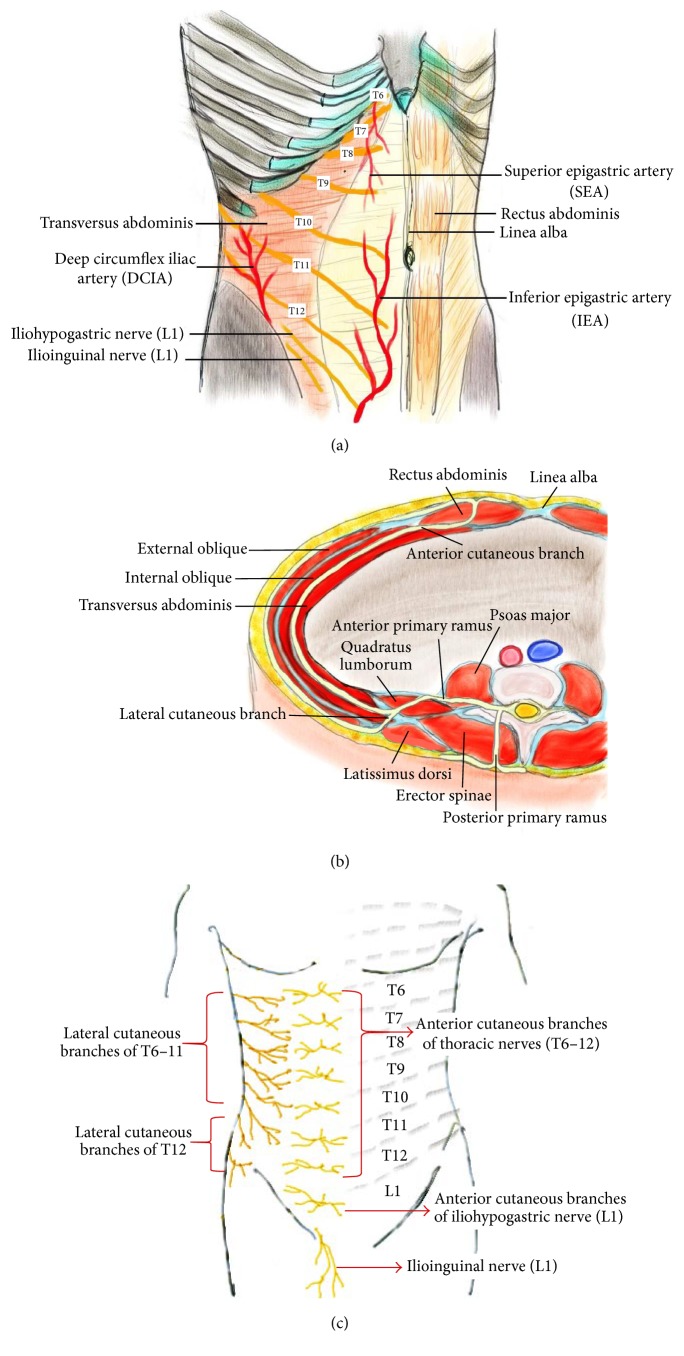
The thoracolumbar spinal nerves (T6~L1) innervating the anterolateral abdominal wall. (a) Distribution of neurovascular structure in the anterolateral abdominal wall. (b) The pathway of the thoracolumbar spinal nerves (T12). This is the cross-sectional view of the left abdomen. The anterior primary ramus of the segmental nerves divides into anterior and lateral cutaneous branches, which supply the anterolateral abdominal wall. (c) The segmental distribution of cutaneous nerve on the anterolateral trunk.

The relevant anatomy is shown in Figure 1. A thorough understanding of the anatomy may help clinicians to determine the site of injection, improve the success rate, and prevent complications.

### 2.1. The Sensory Nerves Innervating the Anterolateral Abdominal Wall

The thoracolumbar nerves are responsible for the segmental cutaneous supply of the abdominal wall. They divide into the anterior primary ramus and posterior primary ramus shortly after exiting from the intervertebral foramen. The posterior ramus travels backward, while the anterior ramus branches into lateral and anterior cutaneous nerves (Figure 1(b)). The anterolateral abdominal wall is mainly innervated by the anterior rami of the thoracolumbar spinal nerves (T6-L1), which become the intercostal (T6-T11), subcostal (T12), and ilioinguinal/iliohypogastric nerves (L1) (Figure 1(a)). These branches further communicate at multiple locations, including large branch communications on the anterolateral abdominal wall (intercostal/upper TAP plexus) and plexuses that run with the deep circumflex iliac artery (DCIA) (lower TAP plexus) and the deep inferior epigastric artery (DIEA) (rectus sheath plexus) [2]. Since these segmental nerves communicate just above the transversus abdominis muscle, the subfascial spread of local anesthetic can provide anterolateral abdominal wall analgesia [23].

### 2.2. Clinical Correlation of Cutaneous Branches

The anterior primary rami of T7-T12 spinal nerves pass between internal oblique and transversus abdominis and then perforate rectus abdominis and end as the anterior cutaneous branches, which innervate the anterior abdomen (from midline to midclavicular line). Among these anterior rami, the T12 crosses quadratus lumborum before entering the TAP, as shown in Figure 1(b) [24]. The lateral cutaneous branches depart near the angle of the rib posteriorly [15]. The lateral cutaneous branches of T7-T11 then divide into anterior and posterior branches: the anterior branches supply the abdominal wall toward the lateral margin of rectus abdominis; the posterior branches pass backward to supply the skin over latissimus dorsi. However, the lateral cutaneous branch of T12 does not further divide into anterior and posterior branches (Figure 1(b)). It supplies a part of the gluteal region, and some of its filaments extend as low as the greater trochanter (Figure 1(c)). The L1 spinal nerve divides into the iliohypogastric and ilioinguinal nerves, which innervate the skin of the gluteal region behind the lateral cutaneous branches of T12, the hypogastric region, the upper medial part of the thigh, and the genital area [25].

Since the lateral cutaneous branches leave the TAP posterior to the midaxillary line, posterior injection of local anesthetics is suggested if analgesia for both the anterior and lateral abdominal wall is required [26]. However, most of the lateral cutaneous branches arise before the main nerves enter the TAP, and only those of T11 and T12 have a short course within or through the TAP [15]. For the blockade of the lateral cutaneous branches, a TAP block can only cover the T11 and T12 lateral cutaneous branches even with a more posterior injection. Based on the distribution of the T9-T12 branches, the lateral approach performed at the midaxillary line between the costal margin and iliac crest could provide mainly periumbilical and infraumbilical analgesia, while the posterior approach performed posterior to the midaxillary line has the potential to provide some degree of lateral abdominal wall analgesia [10]. Paravertebral spread from T5 to L1 has been reported only with posterior TAP blocks [27]. The L1 branches, which become the ilioinguinal and iliohypogastric nerves, pass into the TAP near the anterior part of the iliac crest [15]. Thus, a TAP block at this level is similar to ilioinguinal and iliohypogastric nerve blocks. Direct ilioinguinal/iliohypogastric nerve block is a better choice than TAP block if only L1 analgesia is needed [28, 29].

The spread of injectate in TAP might be affected by anatomical variation [30], injected volume [31], and choice of approach [32–35]. To achieve the best quality of analgesia without increasing the volume and associated systemic toxicity, it is important to choose the most appropriate method by considering the distribution of segmental nerves.

### 2.3. The TAP Block-Related Muscles

**Figure 2:**
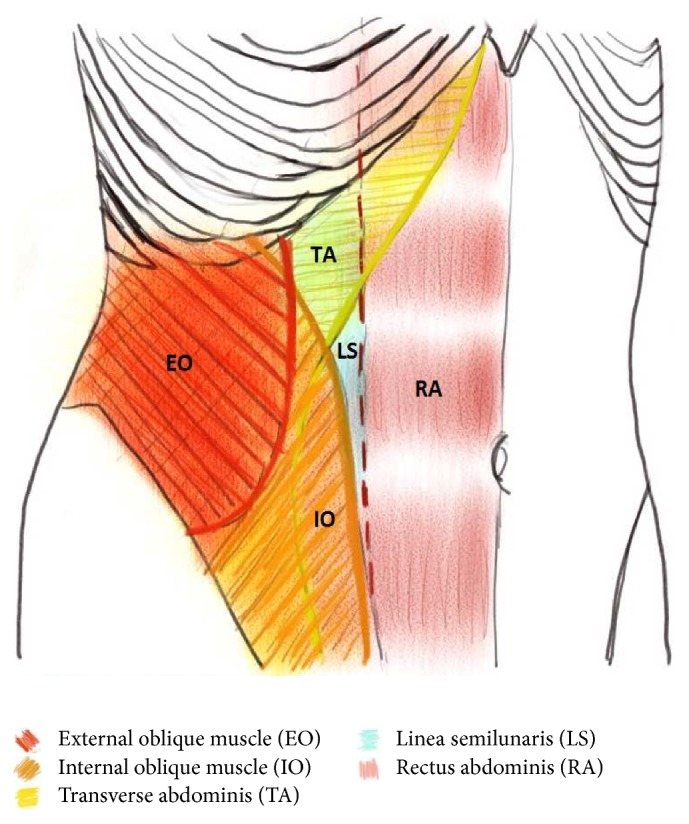
The muscular structure of the anterolateral abdominal wall. RA: rectus abdominis; TA: transversus abdominis; IO: internal oblique; EO: external oblique; LS: linea semilunaris. The red dotted line: the lateral border of rectus abdominis.

There are four paired muscles in the anterolateral abdominal wall: rectus abdominis, transversus abdominis, internal oblique, and external oblique. Rectus abdominis runs parallel in the midline and is separated by the linea alba. The other three are laterally located muscles, transversus abdominis, internal oblique, and external oblique, sequentially from deep to superficial, and are mainly related to TAP blocks. The three muscles overlie one another in the lateral abdomen and terminate medially as an aponeurosis called the linea semilunaris, which is lateral to rectus abdominis [15] (Figure 2). The TAP plexuses lie on transversus abdominis. Therefore, intramuscular injection of local anesthetics might also have some analgesic effects [36].

## 3. New Nomenclature

The TAP is a potential anatomical space between transversus abdominis and internal oblique (or rectus abdominis) [37], and the field block by TAP infiltration is referred to as a TAP block. There are several different approaches for ultrasound-guided TAP block, such as lateral, posterior, and subcostal approaches. Unlike specific peripheral nerve blocks, TAP block is a nondermatomal “field block.” This has led to a debate on whether there is a need for standardization of techniques or technique nomenclature [33]. Even with the same ultrasound-guided technique, the extent of spread of local anesthetics can be variable due to individual anatomical variations [30, 33]. However, there has been evidence supporting the idea that the nuances of various techniques can also affect the analgesic outcomes. For example, a meta-analysis showed that posterior approach appears to produce longer analgesia compared to that of the lateral approach [10]. Furthermore, based on cadaveric and radiologic evaluations, dye injected via different approaches demonstrated different nerve involvement [23, 32, 34, 38]. Therefore, it is important to classify the “TAP block” group according to a reasonable nomenclature system before comparing the analgesic effects among different approaches.

The nomenclature regarding TAP block is confusing, and there is still no consensus about its terminology after an explosive growth in numbers of studies about it. Therefore, we provided a nomenclature system to categorize the various approaches into four groups comprising subcostal, oblique subcostal, lateral, and posterior TAP blocks. The classification is based on the involved spinal nerves rather than the probe positions only. Although all anterior branches communicate on TAP, each segmental nerve supplies different areas (Figure 1(a)). The T6-8 supply the area below the xiphoid and parallel to the costal margin; T9-12 supply the periumbilical area and the lateral abdominal wall between the costal margin and iliac crest; L1 supplies the anterior abdomen near the inguinal area and thigh [15].

Classification of TAP blocks based on a unified nomenclature system is shown in Table 1. Many approaches have been suggested to provide analgesia over the upper abdomen, such as oblique subcostal, subcostal, or upper subcostal approaches [11, 13, 15, 17, 39, 40, 43]. However, they are quite similar in the area where local anesthetics deposit except for the oblique subcostal approach, which covers both the upper and lower abdomen using the hydrodissection technique. We suggest categorizing similar approaches as “subcostal” since it is easier to remember it by probe position and associated blocked plexus.

A midaxillary or lateral TAP block is performed by placing the probe at or anterior to the midaxillary line between the costal margin and iliac crest. It can provide lower abdominal wall analgesia from the midline to the midclavicular line [10, 26]. Compared to a lateral TAP block, a posterior TAP block approximates the double-pop TAP technique at the lumbar triangle of Petit [44] by injecting local anesthetic superficial to the transversus abdominis aponeurosis [45] and offers better and more prolonged analgesia than the lateral approach [10, 42]. While subcostal and lateral TAP injections do not always cover the lateral cutaneous branches of the segmental nerves [35], the posterior approach deposits the injectate posterior to the midaxillary line and may provide better analgesia to the lateral abdominal wall [26].

Dual TAP block, which technically combines subcostal with lateral/posterior TAP block, provides a wider coverage for both the upper and lower abdominal walls. By anesthetizing both the upper TAP plexus (the intercostal plexus, which consists of large branch communications anterolaterally) and the lower TAP plexus (the deep circumflex iliac artery plexus) (Figure 1(a)), a lateral-to-medial long-needle approach can cover T7/8 to L1. [35, 46]. If the dual TAP block is performed bilaterally, it is called bilateral dual TAP block, which was introduced by Borglum et al. [47, 48]. It is similar to the four-quadrant TAP block by Niraj et al. [12, 16]. As Borglum et al. described previously, “dual” stands for two extent areas of the anatomical TAP and expresses the anterior abdominal wall correctly rather than “four-quadrant” one [46]. A TAP block can be performed unilaterally or bilaterally. Therefore, “dual TAP block, unilateral or bilateral,” is more precise and suitable for clinical communication.

As mentioned earlier, the oblique subcostal TAP block is a modified subcostal TAP block, which was first introduced by Hebbard et al. [15]. By hydrodissecting the TAP along the oblique subcostal line (from the xiphoid toward the anterior part of the iliac crest), the anesthetic solution spreads across the location of T6-L1 nerves and thus potentially covers both the upper and lower abdominal walls. Since it requires only a single penetration through the subcostal approach but covers both the upper and lower TAP plexuses like a dual TAP block, it cannot be classified into either one of these two groups appropriately. Thus, the oblique subcostal TAP block should be categorized as an independent, specific technique for TAP block (Table 1). This nomenclature is slightly different from the one proposed by Hebbard [49], which divided the subcostal TAP block to upper subcostal and lower subcostal TAP blocks. Since a lower subcostal TAP block covers the same area as a lateral TAP block and does not provide analgesia over the T7-8 dermatomes, we suggest categorizing the lower subcostal TAP block as a lateral TAP block to simplify the nomenclature. Furthermore, the upper and lower TAP blocks suggested by Borglum et al. correspond exactly to subcostal and lateral approaches, respectively [46].

In addition to the above dichotomy, a posterior TAP block has different manifestations compared to a lateral TAP block, including analgesic effectiveness and duration [10, 42]. Neither a lateral nor subcostal approach results in dye spread posterior to the midaxillary line and thus spares the lateral cutaneous nerve branches, which could possibly be circumvented by the posterior approach [35]. The L1 branches divide into the ilioinguinal and iliohypogastric nerves. If analgesia over the L1 dermatome is the major concern, it is recommended to target the L1 branches specifically. The ilioinguinal and iliohypogastric nerve block can provide more specific and better analgesia than a TAP block [28, 29]. The anterior quadratus lumborum block is also a promising alternative to block the L1 branches coursing over the surface of quadratus lumborum [45]. Ultrasound-guided transversalis fascia plane block also provides analgesia over the L1 dermatome [50]; however, the injection is deeper than TAP blocks and is at risk for unanticipated motor weakness due to central and proximal spread toward psoas major [51].

As described above, classification based on the logic of this nomenclature system is reasonable and clinically useful and can aid in discussion among clinicians. The detailed definition of different approaches will be described in the Techniques of TAP Block.

## 4. Techniques of TAP Block

**Figure 3:**
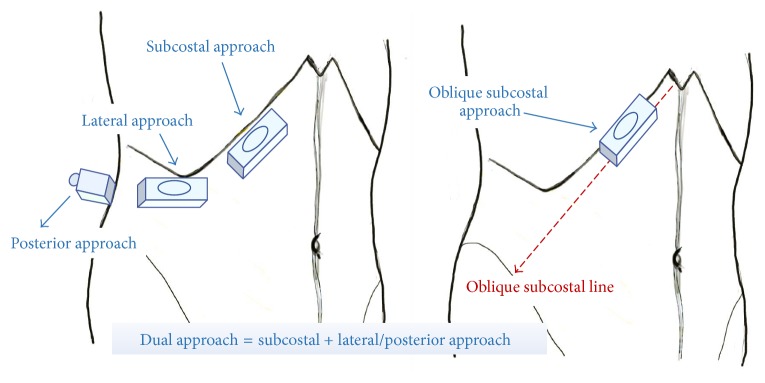
Four approaches of ultrasound-guided transversus abdominis plane (TAP) blocks. Red dashed line indicates the oblique subcostal line, from the xiphoid to the anterior part of the iliac crest.

In this review, we described the original landmark-guided technique in brief and four ultrasound-guided TAP blocks according to the unified nomenclature system: lateral, posterior, subcostal, and oblique subcostal TAP blocks (Table 1 and Figure 3). Furthermore, current advancement in continuous techniques to overcome the limitation of one-shot TAP blocks was discussed. The patient is placed in a supine position for all these approaches, except for slight lateralization for the posterior approach in some cases.

### 4.1. Landmark-Guided TAP Block

The blunt landmark-guided technique applies loss of resistance as the needle is advanced through the fascia layers of external oblique and internal oblique [1]. After locating the triangle of Petit, the TAP is identified using the subjective double-pop loss of resistance technique. McDonnell et al. suggested that the first pop indicates penetration of the fascia of the external oblique muscle, and the second indicates piercing of the fascia of internal oblique and entry of the needle into the TAP [23, 33]. However, Rafi et al. suggested that the first pop indicates the needle has reached the plane between internal oblique and transversus abdominis, and the second pop indicates the needle has passed through transversus abdominis and thus the needle went too far [1, 37]. Debates continue regarding the adequacy of “single-pop” [1], “double-pop” [23], and the structures responsible for the “pop.”

Currently, landmark-guided technique is no longer recommended because of ambiguity of the standard procedure sequence, small size and large variation of the lumbar triangle of Petit, and the risk of peritoneal perforation during the blind technique [37, 52].

### 4.2. Ultrasound-Guided TAP Blocks

Ultrasound guidance is now considered the gold standard for peripheral nerve block [53]. Usually, a linear probe is adequate for most TAP blocks. However, a convex probe is preferable for TAP blocks in markedly obese patients [54, 55].

#### 4.2.1. Ultrasound Identification of TAP

**Figure 4:**
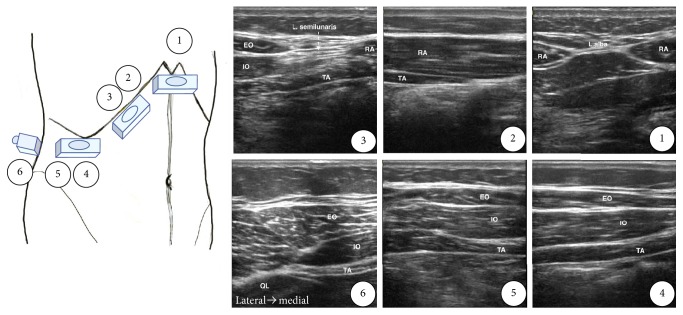
Ultrasound identification of the transversus abdominis plane. RA: rectus abdominis; TA: transversus abdominis; IO: internal oblique muscle; EO: external oblique muscle; QL: quadratus lumborum; L. alba: linea alba; L. semilunaris: linea semilunaris.

To perform an ultrasound-guided TAP block, identification of the TAP is a priority. We suggest the scanning steps as follows: (1) Put the transducer transversely just below the xiphoid process and locate the paired rectus abdominis and the linea alba. (2) Rotate the transducer obliquely and move laterally, parallel to the costal margin. At this level, the TAP is between rectus abdominis and transversus abdominis, or the TAP is absent here because transversus abdominis ends at the lateral end of rectus abdominis in some patients. (3) Move the transducer along the costal margin more laterally until the aponeurosis of the linea semilunaris, which is lateral to the rectus abdominis, appears. Internal oblique and external oblique are located lateral to the linea semilunaris. We can start to identify the three muscle layers: transversus abdominis, internal oblique, and external oblique (from deep to superficial). The TAP is located just above transversus abdominis. (4) Move the transducer more laterally to the midaxillary line, and scan up and down between the costal margin and iliac crest. Typically, three muscle layers can be seen. The TAP is between internal oblique and transversus abdominis. (5) If the transducer is placed posteriorly, we find that internal oblique and transversus abdominis taper off into a common aponeurosis, also called the thoracolumbar fascia, which is connected to the lateral border of the quadratus lumborum. The TAP is between internal oblique and transversus abdominis and continuous with the aponeurosis [40, 56]. The probe position of each ultrasound-guided TAP block is shown in Figure 3, and the corresponding ultrasound images are shown in Figure 4.

#### 4.2.2. Subcostal TAP Block

**Figure 5:**
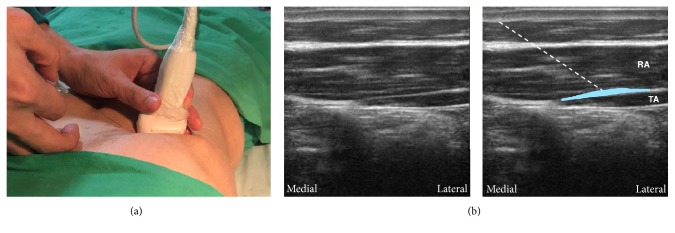
Subcostal approach of transversus abdominis plane (TAP) block. (a) The probe position and needle direction. The probe is parallel to the costal margin near the xiphoid. The needle is inserted in plane. (b) The corresponding ultrasound images. The TAP is between rectus abdominis and transversus abdominis, and the local anesthetic is deposited in this plane to cover the upper TAP plexus. White dashed line: the needle trajectory. Light blue area: the deposition sites of local anesthetic. RA: rectus abdominis; TA: transversus abdominis.

As shown in Figure 5(a) and described in steps (1) and (2), transversus abdominis is identified as the more hypoechoic muscle layer just beneath rectus abdominis. Deposition of the local anesthetic starts between transversus abdominis and rectus abdominis, medial to the linea semilunaris (Figure 5(b)). If transversus abdominis ends at the lateral end of rectus abdominis, the local anesthetic can be deposited between transversus abdominis and internal oblique lateral to the linea semilunaris, but it might be better to include the injection from beneath rectus abdominis toward the lateral side to achieve a higher success rate.

Shibata et al. suggested that only lower abdominal surgery should be an indication for lateral TAP block because of the limited level of sensory block [57]. Hebbard et al. also demonstrated that the lateral TAP block is suitable for surgery below the umbilicus, while the subcostal TAP block is more suitable for supraumbilical and periumbilical analgesia [15]. Lee et al. further proved that there was a difference in the dermatomal spread between lateral and subcostal approaches [41]. The pattern of spread differs depending on the site of injection and it has important implications for the extent of analgesia produced with each approach [27]. Therefore, the subcostal approach should be considered for upper abdominal analgesia.

#### 4.2.3. Lateral TAP Block

**Figure 6:**
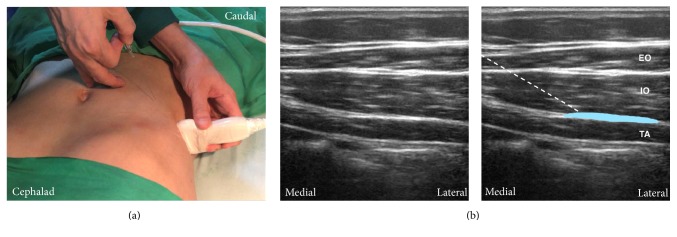
Lateral approach of transversus abdominis plane (TAP) block. (a) The probe position and needle trajectory. The probe is near or at the midaxillary line between the costal margin and the iliac crest. The needle is inserted in plane. (b) Corresponding ultrasound images. The TAP is between internal oblique and transversus abdominis. The local anesthetic is deposited in this plane to cover the lower TAP plexus. White dashed line: needle trajectory. Light blue area: the deposition site of local anesthetic. TA: transversus abdominis; IO: internal oblique; EO: external oblique.

In step (4), we can identify the typical three muscles layers at the midaxillary line between the costal margin and iliac crest. After measuring the depth of the TAP, a needle is inserted away from the transducer at the same distance according to the principle to make the needle in plane for deep regional blocks [58] (Figure 6(a)). The needle is advanced into the transversus abdominis and pulled back incrementally with regular aspiration and then the plane is hydrodissected until the eye sign, an elliptical, hypoechoic spread of local anesthetic, is seen. Otherwise, it is also logical to deposit local anesthetic underneath the fascial layer to ensure optimal analgesia because the nerves are bound to the transversus abdominis [33]. If a patchy opacity appears within the internal oblique, indicating intramuscular injection, or the local anesthetic does not separate the fascia well, the needle tip should be repositioned. However, intramuscular injection of the transversus abdominis might still provide some analgesic effects [36]. Half-the-air setting can also help identify the correct fascial plane using test volume injection and prevent incidental neurologic injury [59, 60].  Figure 6(b) shows the ultrasound image of a lateral TAP block.

#### 4.2.4. Posterior TAP Block

**Figure 7:**
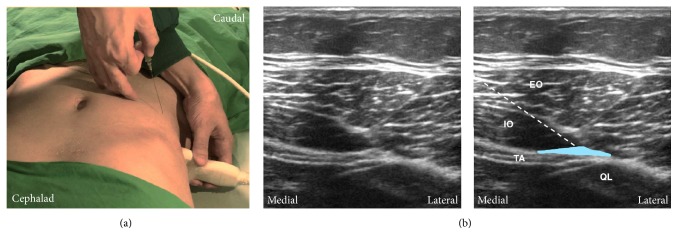
Posterior approach of transversus abdominis plane (TAP) block. (a) The probe position and needle trajectory. The probe is placed posterior to the midaxillary line between the costal margin and the iliac crest. The needle is inserted in plane. (b) Corresponding ultrasound images. Posteriorly, transversus abdominis tails off and turns into the aponeurosis. The quadratus lumborum can be seen posteromedial to the aponeurosis. The injection site is at the TAP between internal oblique and transversus abdominis posterior to the midaxillary line and near the aponeurosis. White dashed line: needle trajectory. Light blue area: the deposition site of local anesthetic. TA: transversus abdominis; IO: internal oblique; EO: external oblique; QL: quadratus lumborum.

The posterior approach is similar to the lateral approach, but the ultrasound transducer is moved more posteriorly as shown in Figure 7(a). This is to view the point where transversus abdominis ends, as described in step (5). When scanning posteriorly, transversus abdominis tails off and turns into the aponeurosis. Quadratus lumborum can be seen posteromedial to the aponeurosis (Figure 7(b)). The injection site is superficial to the aponeurosis near quadratus lumborum [27, 45]. There have been studies suggesting that a posterior TAP block provides more effective and prolonged analgesia than the lateral approach [10, 42]. Evidence showed the absence of posterior spread in the lateral approach [26] and a wider expansion of local anesthetics in the posterior approach [27].

#### 4.2.5. Oblique Subcostal TAP Block

The oblique subcostal TAP block is modified from the subcostal TAP block, which was first introduced by Hebbard et al. [15]. Unlike other approaches, a much longer needle (15–20 cm) and a larger volume of anesthetics (40–80 ml) are required. The oblique subcostal line extends from the xiphoid toward the anterior part of the iliac crest and potentially covers the T6-L1 nerves in the TAP (Figure 3). Thus, local anesthetic injected in the TAP along this line provides both upper and lower abdominal wall analgesia, like a dual TAP block. Compared to a dual TAP block, the oblique subcostal TAP block more consistently covers L1 dermatome. Only single penetration is required for the oblique subcostal approach. A large volume of local anesthetics is required to hydrodissect the TAP along the whole ipsilateral oblique subcostal line. It can provide promising analgesia for abdominal surgeries [61–63] and might be better compared to the lateral approach [64]. However, the oblique subcostal TAP block is much more difficult. Bending the needle initially and then reinserting during the advancement of the needle might be helpful in performing the block [15].

## 5. Other Considerations

### 5.1. Dual TAP Block

If analgesia is needed for both the supraumbilical and infraumbilical abdomen, the dual TAP block could also be considered. Dual TAP block is the combination of the subcostal and the lateral/posterior TAP block. Compared to the oblique subcostal TAP block, the dual TAP block technically ensures more easily that local anesthetic is deposited throughout the plane and provides analgesia for both the upper (T6-T9) and lower (T10-T12) abdomen. The bilateral dual TAP block was first introduced by Borglum et al. as the four-point approach [47]. Niraj et al. once called it the “four-quadrant” TAP block [12]. After making the skin aseptic, we suggest performing the lateral/posterior approach first and then the subcostal approach, to keep the probe aseptic. In other words, the probe is placed in the gravity-dependent part as a general rule below the needle insertion site for single-shot peripheral nerve blocks [65, 66]. Jelly introduction into the central part of the body should be avoided whenever possible, even if it is aseptic [67], and ultrasound gel itself near peripheral nerves may cause inflammation [68]. Performing the dual TAP block in this sequence keeps the needle away from gravity-dependent gel contamination.

### 5.2. Continuous TAP Block

Petersen et al. [69] reported that anesthetized dermatomes produced by a continuous TAP block employing the lateral approach comprised only two segments (T10 and T11) in healthy volunteers. Nevertheless, two previous randomized controlled trials [11, 17] have reported that adding continuous TAP blocks to single-injection TAP blocks improves analgesia after laparotomy for gynecological cancer. Both studies employed an oblique subcostal approach for a continuous TAP block [15]. After incremental hydrodissection of the TAP along the oblique subcostal line, a catheter is threaded through the needle into the TAP. Yoshida et al. [17] proposed that this thorough hydrodissection of the TAP and the catheter passage might facilitate a wider spread of sensory block by providing a track for the local anesthetics along the catheter within the TAP. However, this hypothesis should be validated in a future study. In the two above-mentioned studies regarding continuous oblique subcostal TAP blocks [11, 17], a point-source catheter, such as an epidural catheter, was used for providing a continuous TAP block. A continuous TAP block using a catheter with more extensive holes may produce a wider spread of sensory block and superior analgesia [13], although there has been no research evaluating the effectiveness of the multihole catheter compared to the point-source catheter.

## 6. Complications

Visceral damage due to inadvertent peritoneal puncture while performing blind TAP block has been reported [70]. Although the risk can be minimized with ultrasound guidance, the potential of iatrogenic injury still exists due to a failure to image the entire needle during its advancement [71]. Other reported complications of TAP block include seizure, ventricular arrhythmia, and transient femoral nerve palsy [72–75]. To limit local systemic toxicity, a low concentration of local anesthetic should be chosen when a high-volume regimen (e.g., 20 ml bilaterally) is necessary for a successful block [76]. Good communication between anesthesiologists and surgeons also helps prevent overdose by incidental repeated local anesthetics injection after a TAP block. The immediate availability of lipid emulsion along with other emergency therapeutics is recommended for TAP block [77]. Transient femoral palsy after TAP block is induced by incorrect local anesthetic deposition between transversus abdominis and the transversalis fascia [75]. Since the femoral nerve lies in the same tissue plane, as little as 1 ml of injectate flowing posteromedially can surround the femoral nerve [78]. This complication is usually self-limited but will delay patient discharge especially in day-case surgeries. Using a test solution to locate the needle tip under ultrasound guidance will help identify the TAP and avoid spread of the anesthetic toward the femoral nerve [78].

Since the role of a nerve stimulator during TAP block is elusive and the nervous structures might be too small to be identified by ultrasound, “half-the-air” setting should be considered to avoid intrafascicular spread by keeping the injection pressure below 15 psi [60]. Intrafascicular needle placement associated with high injection pressure can result in neurologic injury in animal models [79, 80]. Monitoring and limiting injection pressure to 15 psi reliably detects needle-nerve contact [81]. Since the TAP belongs to a vessel-rich plane [37], the test solution instead of local anesthetic should be injected first. By using the test solution to hydrolocate the needle tip and visualize the hypoechoic spread, the surrounding tissues, not only vessels but also nerves, are usually pushed away from the needle tip by the test spread [59].

In brief, half-the-air setting takes advantage of the test solution and pressure monitoring at the same time [59]. To avoid all complications mentioned above, it is recommended to inject the least volume of local anesthetic required under dual guidance with ultrasound and half-the-air setting.

## 7. Conclusion

With the advancement in ultrasound technology, the success rate and safety of TAP blocks have markedly improved. There are several different approaches for ultrasound-guided TAP block, and the nuances of various techniques can affect the analgesic outcomes. It is important to classify the “TAP block” group according to a reasonable nomenclature system before comparing the analgesic effects among different approaches. In this review, we provided a nomenclature system to categorize the various approaches into four groups comprising subcostal, lateral, posterior, and oblique subcostal TAP blocks. This new nomenclature system based on the involved spinal nerves is clinically useful and can aid in discussion among clinicians. A posterior TAP block offers a longer duration of analgesia than does a lateral TAP block for the infraumbilical abdominal wall. If analgesia over the supraumbilical wall is required, subcostal, oblique subcostal, or dual TAP blocks are recommended. Adding continuous TAP block to single-injection TAP block can further improve and prolong its analgesic effect. Based on the accumulating evidence, dual guidance with ultrasound and half-the-air setting should be considered for TAP blocks.

## Figures and Tables

**Table 1:** The classification of ultrasound-guided TAP blocks and the corresponding supplied areas.

Approach	The main segmental thoracolumbar nerves [15]		Supplied area [15]
Subcostal [39–41]	T6-9	Anterior cutaneous branches	Upper abdomen just below the xiphoid and parallel to the costal margin

Lateral [10, 26]	T10-12	Anterior cutaneous branches	Anterior abdominal wall at the infraumbilical area, from midline to midclavicular line

Posterior [10, 42]	T9-12	Anterior cutaneous branches (possibly lateral cutaneous branches)	Anterior abdominal wall at the infraumbilical area and possibly lateral abdominal wall between costal margin and iliac crest

Oblique subcostal [11, 13, 15, 17, 43]	T6-L1	Anterior cutaneous branches	Upper and lower abdomen

TAP: transversus abdominis plane.
